# Cognitive profile in multiple sclerosis and post-COVID condition: a comparative study using a unified taxonomy

**DOI:** 10.1038/s41598-024-60368-0

**Published:** 2024-04-29

**Authors:** Cristina Delgado-Alonso, Alfonso Delgado-Alvarez, María Díez-Cirarda, Silvia Oliver-Mas, Constanza Cuevas, Paloma Montero-Escribano, Ana Maria Ramos-Leví, María José Gil-Moreno, Juan Ignacio López-Carbonero, Bruce P. Hermann, Jorge Matias-Guiu, Jordi A. Matias-Guiu

**Affiliations:** 1 Department of Neurology, Hospital Clínico San Carlos, Instituto de Investigacion Sanitaria “San Carlos” (IdISSC), C/Profesor Martín Lagos, 28040 Madrid, Spain; 2 Department of Endocrinology, Hospital Clínico San Carlos, Instituto de Investigacion Sanitaria “San Carlos” (IdISSC), Madrid, Spain; 3 Department of Neurology, School of Medicine and Public Health, University of Wisconsin, Madison, WI USA

**Keywords:** Multiple sclerosis, COVID-19, Cognition, Fatigue, Long COVID, Multiple sclerosis, Central nervous system infections

## Abstract

Post-COVID condition (PCC) and multiple sclerosis (MS) share some clinical and demographic features, including cognitive symptoms and fatigue. Some pathophysiological mechanisms well-known in MS, such as autoimmunity, neuroinflammation and myelin damage, have also been implicated in PCC. In this study, we aimed to compare the cognitive phenotypes of two large cohorts of patients with PCC and MS, and to evaluate the relationship between fatigue and cognitive performance. Cross-sectional study including 218 patients with PCC and 218 with MS matched by age, sex, and years of education. Patients were evaluated with a comprehensive neuropsychological protocol and were categorized according to the International Classification of Cognitive Disorders system. Fatigue and depression were also assessed. Cognitive profiles of PCC and MS largely overlapped, with a greater impairment in episodic memory in MS, but with small effect sizes. The most salient deficits in both disorders were in attention and processing speed. The severity of fatigue was greater in patients with PCC. Still, the correlations between fatigue severity and neuropsychological tests were more prominent in the case of MS. There were no differences in the severity of depression among groups. Our study found similar cognitive profiles in PCC and MS. Fatigue was more severe in PCC, but was more associated with cognitive performance in MS. Further comparative studies addressing the mechanisms related to cognitive dysfunction and fatigue may be of interest to advance the knowledge of these disorders and develop new therapies.

## Introduction

Cognitive dysfunction and fatigue are commonly reported after the acute phase of SARS-CoV-2 infection and have been emphasized as the most frequent symptoms by the World Health Organization in the post-COVID condition or Long-COVID (PCC)^1^. Several studies have confirmed the presence of objective cognitive deficits in neuropsychological assessments^2,3^. PCC occurs mainly in middle and working age, and women are predominant^4–7^. Cognitive deficits are more prominent in attention and processing speed, episodic memory and executive function and have been linked to structural and functional brain changes in neuroimaging studies^8–13^. A longitudinal study showed greater reductions in cortical thickness and brain volumes in patients after COVID-19 than in healthy controls compared with neuroimaging acquired before the pandemic^14^. A recent study has also associated fatigue in PCC with structural imaging changes in the thalamus and basal ganglia^15^.

Similarly, most patients with Multiple Sclerosis (MS) also report fatigue and cognitive deficits. Cognitive deficits are especially focused on attention and processing speed impairment, followed by executive function and episodic memory. MS is a recognized autoimmune disorder, and cognitive deficits have been linked to cortical and subcortical structural and functional brain damage^16,17^.

Although the pathophysiology of PCC and the neurological symptoms of PCC is still unknown, several studies suggest mechanisms of neuroinflammation, autoimmune disorders, myelin dysregulation, and reactivation of another virus (such as Epstein-Barr infection)^18,19^. Although in a different clinical course and extent, these mechanisms are at least partially shared with MS. Besides, the role of Epstein-Barr virus or other viral infections in the development of MS and/or disease activity is supported by some studies^20^. Overall, this suggests an interest in evaluating the similarities and differences in the cognitive profiles of patients with PCC and MS. Comparative studies may also be useful to contextualize the cognitive deficits found in PCC, which have important socioeconomic consequences^21^. However, to our knowledge, there are no studies comparing cognitive dysfunction associated with PCC and MS. In addition, the relationship between fatigue and cognitive performance is still unclear. Previous studies in MS have found that cognitive tests assessing vigilance and alertness are more related to fatigue, which could be caused by shared mechanisms associated with brain atrophy and neurochemical dysfunction^22^. In PCC, fatigue has also correlated with some attentional tests^23,24^. Thus, this study aimed to compare the cognitive phenotypes of two large cohorts of patients with PCC and MS that were examined with the same neuropsychological protocol. We also aimed to evaluate the relationships between fatigue, and cognitive performance in the two cohorts. We also compared the frequency of depression, which is a relevant factor in both MS and PCC^25–27^.

## Methods

### Study design and participants

We conducted a cross-sectional investigation including patients with PCC and MS involved in previous research studies evaluating the cognitive characteristics of these disorders^28,29^. Patients were recruited from specific clinical programs dedicated to diagnosing and treating individuals with PCS and MS, where comprehensive neuropsychological assessment were integrated into the clinical protocol. The research protocol was approved by the Ethics Committee of our center (Comité de Ética de la Investigación con Medicamentos del Hospital Clínico San Carlos). Written informed consent was obtained from all participants.

Patients with PCC met the following criteria: (a) Diagnosis of COVID-19 confirmed by RT-PCR; (b) cognitive complaints or fatigue in close relationship with SARS-CoV-2 infection; (c) WHO criteria for Post-COVID-19 condition^1^. Exclusion criteria were as follows: (a) any cognitive complaint before COVID-19; (b) any medical, systemic, neurological or developmental comorbidity potentially linked to cognitive dysfunction; (c) history of alcohol or drug abuse; (d) neuropsychiatric disorders not attributable to PCC; (e) any sensory or motor disorder potentially biasing assessments.

Patients with MS met the following criteria: (a) diagnosis of MS according to the 2010 McDonald criteria^30^; (2) age between 18 and 80 years. Exclusion criteria were as follows: (a) a relapse within the previous two months or active treatment with corticosteroids; (b) any other medical, systemic, neurological or developmental comorbidity potentially causing cognitive impairment; (c) history of alcohol or drug abuse; (d) neuropsychiatric disorders not attributable to MS; (e) sensory or motor disorder biasing assessments.

From an initial sample of 240 patients with PCC (mean age 48.42 ± 10.84 years, 77.9% of women, mean time since the acute infection of 17.48 ± 8.43 months), and 298 patients with MS (mean age 48.09 ± 9.84 years, 69.8% of women, mean duration of disease of 15.87 ± 7.85 years), a matched sample of 436 participants (218 per group) was obtained. The main clinical and demographic characteristics of each group and the vaccination status are presented in Table 1. Time of SARS-CoV-2 infection (month and year) leading to PCC and time of assessments is shown in Supplementary Fig. 1.

**Table 1:** Demographic and clinical characteristics.

	PCC (n = 218)	MS (n = 218)	T/X^2^ (*p*-value)
Age	48.28 ± 9.59	48.41 ± 9.50	− 0.14 (0.884)
Sex (% women)	174 (79.8%)	174 (79.8%)	0 (1.0)
Years of education	15.44 ± 3.21	15.39 ± 3.34	0.16 (0.872)
Disease duration^1^	17 ± 8 months	15.87 ± 7.85	–
EDSS^2^	–	3 [1.5–5.0]	–
MS subtype (%)	–	86,9% RR, 5.0% SP, 1.4% PP	–
Hospitalization during the acute infection	44 (20.18%)	–	–
Ventilatory assistance	16 (7.3%)	–	–
Intensive care unit admission	14 (6.4%)	—	—
Vaccinated at the time of being infected by SARS-CoV-2	171 (78.4%)	—	—
Vaccinated at the time of assessment (at least two doses)	8 (3.7%)	—	—
Fatigue	188 (86.2%)	141 (64.7%)	28.4 (< 0.001)
Depression	65 (29.8%)	61 (27.9%)	0.18 (0.672)

Data is reported as absolute frequency (percentage) or mean ± standard deviation. *RR* relapsing–remitting, *SP* secondary progressive, *PP* primary progressive.

^1^Months since the onset of acute infection in PCC and years of disease duration in MS.

^2^EDSS: Expanded disability scale status scale. Values are shown as median [Q1–Q3].

### Neuropsychological and behavioral assessments

Patients were evaluated with a comprehensive neuropsychological protocol that is mainly based on the cognitive tests included in the Neuronorma battery. This was a set of neuropsychological tests co-normed in our country in older and young people^31,32^ and has been validated in several diseases^33^. Previous works by our group implemented this battery to describe the cognitive profile in patients with MS and recently in PCC^28,29^. This battery was administered by trained neuropsychologists. The following tests were shared in the assessment of patients with PCC and MS and were included in the present study: forward and backward digit span, Corsi block-tapping test, Symbol Digit Modalities Test, Boston Naming Test (BNT), Judgment Line Orientation (JLO), Rey-Osterrieth Complex Figure (ROCF) (copy and recall at 3, 30 min, and recognition), Free and Cued Selective Reminding Test (FCSRT) (total free recall, total recall, delayed free recall, and delayed total recall), verbal fluencies (animals and words beginning with “*p*” and “m” in 1 min each one), Stroop Color-Word Interference Test.

Furthermore, patients were evaluated with the Modified Fatigue Impact Scale (MFIS)^34^. MFIS contains 21 items related to cognitive, physical, and psychosocial dimensions of fatigue, which are scored using a Likert-type scale. The assessment evaluates the impact of fatigue in the past 4 weeks. Additionally, depression was assessed with the Beck Depression Inventory-II^32^. Following previous literature, we used a cut-off of ≥ 38 to delineate fatigue and ≥ 19 to define moderate to severe depression^34,35^.

### Statistical analysis

Statistical analysis was conducted using IBM(R) SPSS v26.0, JASP v0.16.1 and R software^36^. Figures were prepared using the ggplot2 package (v3.4.1). Using MedCalc 20.218, patients with MS and PCC were matched 1:1 according to sex, age (± 3 years), and years of education (± 3 years). The two independent samples t-test was used to compare the two groups. Effect sizes were estimated with Cohen’s d, and were classified as small (d = 0.2–0.49), moderate (d = 0.5–0.79), and large (d ≥ 0.8).

We calculated the percentage of impairment of each test according to the normative data correcting by age, years of education, and sex when needed. Normative data are based on a multicenter study conducted in Spain before the pandemic^31,32^. In addition, we used the criteria proposed by IC-CoDiMS and IC-CoDi-COVID groups to describe the cognitive phenotypes in patients with MS and PCC, respectively^37,38^. In this taxonomy, initially developed for epilepsy as IC-CoDE^39,40^, a domain is considered impaired when two tests within the same domain fall below the cutoff. For this study, we used -1 S.D as the cutoff to define impairment, according to the findings of the previous studies in both MS and PCC using these criteria^37,38^. Five cognitive domains are considered: attention/processing speed, executive function, language, visuospatial, and episodic memory. Then, according to the number of domains impaired, the patients are classified as: cognitively intact, single-domain impairment, bi-domain impairment, or multi-domain impairment (≥ 3 impaired domains). The tests specified in Table 2 were used to describe each cognitive domain. The chi-squared test was used to compare cognitive phenotypes between MS and PCC.

**Table 2:** Cognitive domains and neuropsychological tests included representing those domains.

Attention and information processing speed	SDMT, Stroop trial 1
Executive function	Stroop trial 3, Digit span backwards
Episodic memory	FCSRT (total delayed recall), ROCF (memory at 30 min)
Visuospatial	JLO, ROCF (copy accuracy)
Language	Boston Naming Test, Semantic verbal fluency

*FCSRT* free and cued selective reminding test, *JLO* judgment line orientation, *ROCF* Rey-Osterrieth complex figure, *SDMT* symbol digit modalities test.

Pearson’s coefficient was used to estimate the correlations between fatigue and neuropsychological tests in PCC and MS. Coefficients < 0.40 were interpreted as a weak correlation, 0.40–0.69 as moderate, and > 0.69 as strong. Fisher r-to-z transformation was calculated to compare between correlation coefficients.

A *p*-value < 0.05 was considered statistically significant. Due to the number of cognitive tests in the neuropsychological protocol, we also specified those contrasts statistically significant after False-Discovery Rate (FDR) correction in the comparison of cognitive performance between PCC and MS.

### Ethical approval

This study was approved by the Ethics and Research Committee from our centre and was performed according to the Declaration of Helsinki and its later amendments.

## Results

### Comparison between PCC and MS

Patients with MS showed lower raw scores compared to PCC in Corsi forward and backwards, FCSRT (total free recall), ROCF memory at 3 and 30 min, and semantic verbal fluency. Conversely, PCC showed greater fatigue severity measured with MFIS. There were no statistically significant differences in the other neuropsychological tests and depressive symptoms. Effect size was moderate for fatigue, and low for the other significant neuropsychological tests. All results are shown in Table 3.

**Table 3:** Neuropsychological test results (raw scores) for PCC and MS groups.

Test	PCC	MS	t	*p*-value	Cohen’s d
Digit span forward	5.79 ± 1.42	5.82 ± 0.94	– 0.19	0.843	– 0.019
Digit span backward	4.21 ± 1.38	4.15 ± 0.97	0.48	0.632	0.046
Corsi test forward	5.59 ± 1.12	5.36 ± 0.99	2.24	0.025	0.215
Corsi test backward	4.92 ± 1.18	4.60 ± 0.97	3.09	0.002*	0.297
SDMT	43.15 ± 13.28	42.35 ± 14.40	0.60	0.546	0.058
Boston Naming Test	52.73 ± 5.59	52.78 ± 5.25	– 0.08	0.930	– 0.008
ROCF copy accuracy	33.50 ± 3.09	33.12 ± 4.19	1.06	0.287	0.102
ROCF copy (time in seconds)	131.81 ± 53.13	137.24 ± 80.22	– 0.83	0.405	– 0.080
FCSRT free recall 1	7.84 ± 2.29	7.60 ± 2.35	1.10	0.272	0.106
FCSRT total free recall	28.59 ± 7.10	26.74 ± 7.36	2.66	0.008	0.255
FCSRTtotal recall	41.25 ± 7.54	41.19 ± 7.03	0.07	0.938	0.007
FCSRT delayed free recall	10.09 ± 3.40	10.08 ± 3.50	0.01	0.990	0.001
FCSRT delayed total recall	14.14 ± 2.88	14.03 ± 2.98	0.37	0.708	0.036
ROCF (memory at 3 min)	19.74 ± 6.88	17.69 ± 7.42	2.99	0.003	0.287
ROCF (memory at 30 min)	19.39 ± 6.83	17.10 ± 7.29	3.37	< .001*	0.323
ROCF (memory recognition)	8.73 ± 3.06	8.98 ± 2.80	– 0.44	0.660	– 0.042
Stroop trial 1	91.55 ± 23.27	91.00 ± 21.76	0.25	0.798	0.025
Stroop trial 2	63.11 ± 15.06	62.57 ± 14.97	0.37	0.708	0.036
Stroop trial 3	37.19 ± 11.69	37.86 ± 12.81	– 0.56	0.574	– 0.054
Semantic fluency	22.02 ± 5.91	19.99 ± 5.86	3.61	< .001	0.346
Letter fluency (p)	16.09 ± 5.02	15.19 ± 5.40	1.80	0.071	0.173
Letter fluency (m)	13.81 ± 4.64	13.03 ± 4.64	1.74	0.082	0.169
Letter fluency (r)	13.46 ± 4.85	12.82 ± 4.58	1.39	0.164	0.135
JLO	22.35 ± 5.66	23.04 ± 4.48	– 1.40	0.161	– 0.135
MFIS (total score)	56.53 ± 15.51	46.31 ± 23.26	5.38	< .001	0.516
BDI-II	14.60 ± 8.40	13.45 ± 11.48	1.18	0.236	0.114

*BDI-II* beck depression inventory, *FCSRT* free and cued selective reminding test, *JLO* judgment line orientation, *MFIS* modified fatigue impact scale, *ROCF* Rey-Osterrieth complex figure, *SDMT* symbol digit modalities test. statistically significant *p*-values after FDR correction are marked with *.

### Cognitive phenotypes

**Figure 1:**
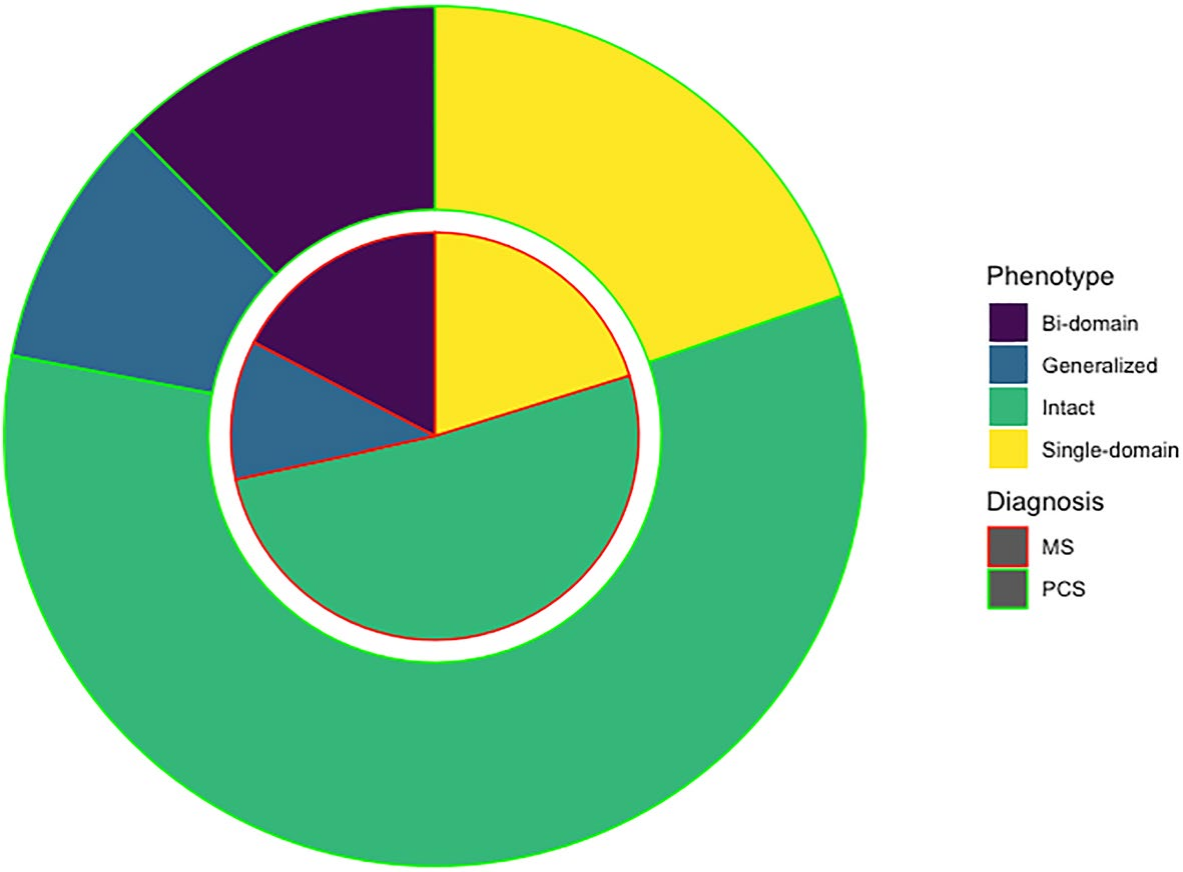
Circle chart representing the cognitive phenotypes in PCC and MS.

There were no statistically significant differences in the cognitive phenotypes (χ^2^ 3.014, *p* = 0.389). Specifically, 127 (58.25%) of PCC patients were regarded as cognitively intact, and 91 (41.74%) as cognitively impaired, 43 (19.72%) showed single-domain, 27 (12.38%) bi-domain, and 21 (9.63%) generalized impairment. Patients with MS were classified as cognitively intact in 112 cases (51.37%), and cognitively impaired in 106 (48.62%). Of those with impairment, 44 (20.18%) showed single-domain, 38 (17.43%) bi-domain, and 24 (11.00%) generalized impairment (Fig. 1).

Regarding the specific cognitive domains, 63 (28.89%) of PCC and 81 (37.15%) of MS showed impairment in attention/processing speed (χ^2^ = 3.36, *p* = 0.067); 24 (11.00%) and 37 (16.97%) in episodic memory (χ^2^ = 3.22, *p* = 0.073); 41 (18.8%) and 46 (21.10%) in executive function (χ^2^ = 0.359, *p* = 0.549); 14 (6.42%) and 16 (7.33%) in visuospatial function (χ^2^ = 0.143, *p* = 0.705); and 22 (10.09%) and 26 (11.92%) in language (χ^2^ = 0.375, *p* = 0.541). The frequency of impairment of each individual test is shown in Supplementary Fig. 2. Patients with MS showed higher frequency of impairment in Stroop trial 1 (χ^2^ = 6.29, *p* = 0.012), semantic fluency (χ^2^ = 9.86, *p* = 0.002), letter fluency (χ^2^ = 9.42, *p* = 0.002), ROCF at 3 and 30 min (χ^2^ = 6.12, *p* = 0.013 and χ^2^ = 13.28, *p* < 0.001, respectively), and FCSRT total free recall (χ^2^ = 7.20, *p* = 0.007). The other tests, including SDMT, showed no statistically significant differences (*p* > 0.05).

### Comparison of cognitive profiles within the groups with cognitive impairment

**Figure 2:**
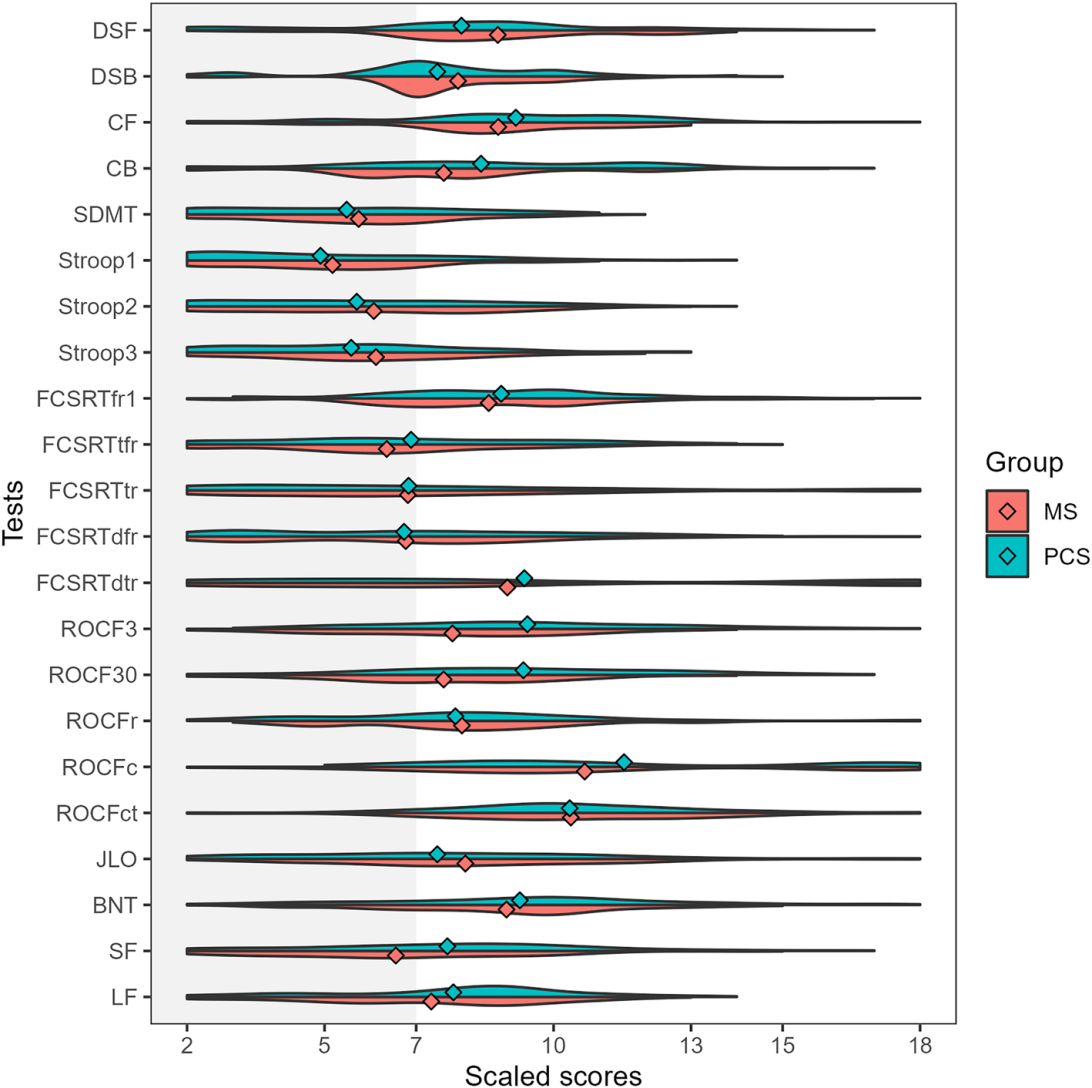
Violin plots representing the adjusted scaled scores (mean 10, standard deviation 3) in each cognitive test in patients with PCC (green) and MS (red) classified as cognitively impaired. The dots represent the mean of each group. *DSF* digit span forward, *DSB* digit span backward, *CF* Corsi forward, *CB* Corsi backward, *SDMT* symbol digit modalities test, *FCSRT* (free and cued selective reminding test, *fr1* free recall 1, *tfr* total free recall, *tr* total recall, *dfr* delayed free recall, *dtr* delayed total recall, *ROCF3* Rey-Osterrieth complex figure (memory at 3 min), *ROCF30* Rey-Osterrieth complex figure (memory at 30 min), *ROCFr* Rey-Osterrieth complex figure (memory recognition), *ROCFc* Rey-Osterrieth complex figure (copy accuracy), *ROCFct* Rey-Osterrieth complex figure (copy time), *JLO* judgment line orientation, *BNT* Boston naming test, *SF* semantic fluency (animals), *LF* letter fluency (words beginning with “*p*”).

We also compared those patients meeting the criteria for cognitive impairment with PCC and MS. Patients with MS showed lower scores in ROCF memory at 3 min and 30 min and semantic fluency in age- and education-adjusted scaled scores (Supplementary Table 1). Patients with PCC showed greater severity of fatigue (59.95 ± 14.98 vs 54.47 ± 20.89, t = 2.13, *p* = 0.034). As depicted in Fig. 2, the represented cognitive profile showed a more prominent impairment in those tests associated with attention and information processing speed.

### Correlations between fatigue and neuropsychological tests

**Figure 3:**
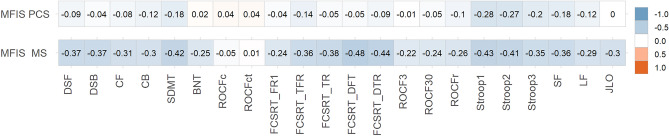
Heatmap showing correlations between MFIS (total score) and neuropsychological tests scores in PCC and MS. The size and direction of the correlation are shown in the right vertical label. *DSF* digit span forward, *DSB* digit span backward, *CF* Corsi forward, *CB* Corsi backward, *SDMT* symbol digit modalities test, *FCSRT* (free and cued selective reminding test), *fr1* free recall 1, *tfr* total free recall, *tr* total recall, *dfr* delayed free recall, *dtr* delayed total recall, *ROCF3* Rey-Osterrieth complex figure (memory at 3 min), *ROCF30* Rey-Osterrieth complex figure (memory at 30 min), *ROCFr* Rey-Osterrieth complex figure (memory recognition), *ROCFc* Rey-Osterrieth complex figure (copy accuracy), *ROCFct* Rey-Osterrieth complex figure (copy time), *JLO* judgment line orientation, *BNT* Boston naming test, *SF* semantic fluency (animals), *LF* letter fluency (words beginning with “*p*”).

All correlations are shown in Fig. 3. In PCC, MFIS (total score) showed weak correlations with SDMT, FCSRT (total free recall and total recall), Stroop (parts 1, 2, and 3) and semantic and letter fluency. In MS, MFIS (total score) showed moderate correlations with SDMT, FCSRT (free delayed recall and total delayed recall), Stroop test (parts 1 and 2); and weak correlations were found with almost all the other tests.

Patients with MS showed higher correlations than PCC in the following tests: digit span forward (z = 3.02, *p* = 0.002), digit span backward (r = 3.53, *p* < 0.001), Corsi forward (Z = 2.45, *p* = 0.014), SDMT (z = 2.82, *p* = 0.004), Boston Naming Test (z = 2.81, *p* = 0.005), FCSRT recall trial 1 (Z = 2.15, *p* = 0.015), FCSRT total free recall (Z = 2.38, *p* = 0.017), FCSRT total recall (z = 3.63, *p* < 0.001), FCSRT delayed free recall (Z = 4.94, *p* < 0.001), FCSRT delayed total recall (Z = 3.93, *p* < 0.001), ROCF (memory at 3 min) (Z = 2.26, *p* = 0.011), ROCF (memory at 30 min) (z = 2.1, *p* = 0.035), semantic fluency (Z = 2.03, *p* = 0.04), letter fluency (M-words) (z = 2.64, *p* = 0.008), letter fluency (R-words) (z = 3.02, *p* = 0.002), Judgment Line Orientation (Z = 3.26, *p* = 0.001). There were no statistically significant differences (*p* > 0.05) in the comparison of correlation coefficients in Stroop (trials 1, 2, and 3), Corsi backward, ROCF (copy accuracy and time), ROCF (memory recognition), and letter fluency (*P*-words).

## Discussion

In this study, we examined the existence of differences in cognitive characteristics between PCC and MS, and the relationship between cognitive function and fatigue. We used two large cohorts of patients that were evaluated with a common neuropsychological protocol. Our study found a significant overlap in cognitive profile between both diseases. Importantly, attention and processing speed were the most pronounced deficits in both disorders, which is consistent with previous studies^2,37,41,42^. Few differences were found in episodic memory tests, which were more impaired in the group of patients with MS than PCC. Similarly, semantic fluency was also more impaired, which could also be linked to the greater impairment of episodic memory^43^. However, effect sizes for these tests were small, confirming that MS and PCC present a very similar cognitive profile.

We applied a novel approach using an international classification of cognitive disorders that is being implemented across several disease groups^37,38,40,44^. This classification system is based on a five-domain cognitive model (attention/processing speed, executive function, episodic memory, visuospatial function, and language) and provides a working definition of impairment to identify cognitive phenotypes and improve cognitive diagnostics. This taxonomy has found reproducible findings across several independent cohorts examined with different neuropsychological batteries in epilepsy^40^, multiple sclerosis^37^ and PCC^38^. It has also shown favorable cross-cultural properties in diverse settings^36^. Our study also supports the use of this taxonomy as a valid method for comparative research across disorders.

By comparing both disorders, the similarities in the cognitive characteristics and the severity of deficits contribute to contextualizing the cognitive dysfunction in PCC. In this regard, our findings suggest that cognitive deficits in PCC are almost as pronounced and prevalent as in MS, and fatigue is even more severe, supporting the mounting evidence that fatigue and cognitive dysfunction are associated with occupational issues and socioeconomic consequences^45,46^.

The severity and frequency of fatigue was greater in patients with PCC. Interestingly, correlations between MFIS total score (evaluating fatigue impact in the last 4 weeks) and neuropsychological tests were larger in the case of MS. However, the cognitive tests that showed stronger correlations with MFIS were similar in both disorders (e.g., Stroop). This may suggest common mechanisms and neural underpinnings in fatigue and cognitive dysfunction in both disorders, as has been recently described^9^. This opens the way to test new therapies for fatigue based on their association with functional brain changes, such as non-invasive brain stimulation, which have shown positive effects in two clinical trials^47,48^. However, at the same time, the lower correlation with neuropsychological tests and the greater severity of fatigue in PCC suggest the existence of other mechanisms (probably not dependent on the central nervous system and including systemic processes such as immune mechanisms, mitochondrial dysfunction or muscle abnormalities) involved in the pathophysiology of fatigue in PCC^24,49,50^. In contrast, fatigue in MS would be more dependent on central mechanisms.

Another interesting result is the lack of significant differences in the severity and frequency of depressive symptoms. Although neuropsychiatric symptoms have been especially emphasized in PCC, most studies did not include a control group^51^. The prevalence of depression is higher in MS than in the general population, and has been associated with several factors, including genetic and immunological factors, brain changes, and psychosocial factors^52^. Similarly, in PCC, proinflammatory factors and psychosocial factors have been hypothesized, but clear evidence about the pathophysiology of depression is still lacking.

Our study has some limitations. First, although the protocol includes several tests of the main cognitive domains, we acknowledge the possibility of differences between groups if other specific tests are used. In this regard, a more thorough analysis of attention and executive function subdomains may be of interest to further characterize the cognitive mechanisms impaired in each disorder. In this study, we selected only those tests shared by both cohorts to avoid potential differences in the frequency of impairment to the length of the battery or the number of neuropsychological tests and scores. Second, fatigue was only assessed with MFIS, which mainly evaluates the impact of fatigue in daily living. More comprehensive questionnaires may be of interest to evaluate potential differences in the clinical characteristics of fatigue across disorders. Additionally, it could also be of interest to evaluate the feeling of fatigue on the same day of the examination because MFIS considers the fatigue severity in the 4 weeks before the assessment. Third, our study is performed in a single center. However, demographic characteristics and degree of impairment in both PCC and MS are consistent with previous studies of the literature, suggesting that both cohorts are representative of these disorders. In this regard, the most important proportion were infected during the first waves (especially the first in March 2020) and before vaccines were available. Furthermore, we must acknowledge the possibility of selection bias, particularly concerning MS, where individuals with more pronounced motor and cognitive impairments may be less inclined to undergo extensive neuropsychological evaluations. Nevertheless, our study was conducted within a framework where comprehensive neuropsychological assessments are standard practice for both MS and PCS. Additionally, the demographic characteristics of our participants closely resemble those of other large-scale studies published in the field^53,54^.

In conclusion, our study finds similar cognitive profiles in PCC and MS, which are mainly characterized by attention and processing speed deficits. Fatigue was more severe in PCC, but the relationship between fatigue and cognitive function was greater in the case of MS. Further comparative studies addressing the mechanisms associated with cognitive dysfunction and fatigue may be of interest to advance the knowledge of these disorders and develop new therapies.

### Supplementary Information

Supplementary Legends.

Supplementary Table 1.

Supplementary Figure 1.

Supplementary Figure 2.

## Data Availability

The datasets generated and analyzed are available from the corresponding author on reasonable request.
