# Supplementary material for: Cognitive profile in multiple sclerosis and post-COVID condition: a comparative study using a unified taxonomy
Source: Sci Rep. 2024 Apr 29;14:9806. doi: 10.1038/s41598-024-60368-0 (PMC11059260; doi:10.1038/s41598-024-60368-0)
Supplement: Supplementary file 1 — Supplementary Legends. [file 41598_2024_60368_MOESM1_ESM.docx]

**Supplementary Figure 1. A)** Time of SARS-CoV-2 infection leading to PCC. **B)** Time of assessment.

**Supplementary Figure 2**. Percentage of impairment (using -1 SD as cutoff) in each neuropsychological test in PCC (in red) and MS (in green).

**Supplementary Table 1**. Neuropsychological test results (scaled-scores) for PCC and MS groups classified as cognitively impaired.
