# Supplementary material for: Cognitive profile in multiple sclerosis and post-COVID condition: a comparative study using a unified taxonomy
Source: Sci Rep. 2024 Apr 29;14:9806. doi: 10.1038/s41598-024-60368-0 (PMC11059260; doi:10.1038/s41598-024-60368-0)
Supplement: Supplementary file 2 — Supplementary Table 1. [file 41598_2024_60368_MOESM2_ESM.docx]

| **Supplementary Table 1**. Neuropsychological test results (scaled scores) for PCC and MS groups classified as cognitively impaired. | | | | | | | | |  |
| --- | --- | --- | --- | --- | --- | --- | --- | --- | --- |
| *Test* | *PCC* | *MS* |  | *t* |  | *p-value* |  |  |  |
| Digit span forward | 7.99 | 8.78 |  | -1.895 |  | 0.060 |  |  |  |
| Digit span backward | 7.46 | 7.92 |  | -1.460 |  | 0.146 |  |  |  |
| Corsi test forward | 9.18 | 8.79 |  | 0.974 |  | 0.331 |  |  |  |
| Corsi test backward | 8.42 | 7.60 |  | 1.923 |  | 0.056 |  |  |  |
| SDMT | 5.48 | 5.75 |  | -0.746 |  | 0.457 |  |  |  |
| Boston Naming Test | 9.26 | 8.97 |  | 0.672 |  | 0.502 |  |  |  |
| ROCF copy accuracy | 11.538 | 10.679 |  | 1.403 |  | 0.162 |  |  |  |
| ROCF copy (time in seconds) | 10.35 | 10.38 |  | -0.062 |  | 0.950 |  |  |  |
| FCSRT free recall 1 | 8.86 | 8.58 |  | 0.688 |  | 0.493 |  |  |  |
| FCSRT total free recall | 6.89 | 6.36 |  | 1.248 |  | 0.213 |  |  |  |
| FCSRTtotal recall | 6.84 | 6.82 |  | 0.024 |  | 0.981 |  |  |  |
| FCSRT delayed free recall | 6.74 | 6.77 |  | -0.080 |  | 0.936 |  |  |  |
| FCSRT delayed total recall | 9.36 | 8.99 |  | 0.428 |  | 0.669 |  |  |  |
| ROCF (memory at 3 minutes) | 9.429 | 7.792 |  | 3.848 |  | <0.001* |  |  |  |
| ROCF (memory at 30 minutes) | 9.34 | 7.60 |  | 4.214 |  | <0.001* |  |  |  |
| ROCF (memory recognition) | 7.86 | 8.00 |  | -0.345 |  | 0.731 |  |  |  |
| Stroop trial 1 | 4.91 | 5.13 |  | -0.607 |  | 0.544 |  |  |  |
| Stroop trial 2 | 5.70 | 6.08 |  | -0.913 |  | 0.362 |  |  |  |
| Stroop trial 3 | 5.58 | 6.12 |  | -1.458 |  | 0.146 |  |  |  |
| Semantic fluency | 7.68 | 6.56 |  | 2.609 |  | 0.010* |  |  |  |
| Letter fluency (p) | 7.81 | 7.33 |  | 1.276 |  | 0.203 |  |  |  |
| Letter fluency (m) | 8.87 | 7.67 |  | 2.426 |  | 0.016* |  |  |  |
| Letter fluency (r) | 8.76 | 8.14 |  | 1.664 |  | 0.098 |  |  |  |
| JLO | 7.46 | 8.08 |  | -1.111 |  | 0.268 |  |  |  |
| MFIS (total score) | 59.95 | 54.47 |  | 2.081 |  | 0.039 |  |  |  |
| BDI-II | 16.65 | 15.53 |  | 0.713 |  | 0.477 |  |  |  |

BDI-II: Beck Depression Inventory; FCSRT: Free and Cued Selective Reminding Test; JLO: Judgment Line Orientation; MFIS: Modified Fatigue Impact Scale; ROCF: Rey-Osterrieth Complex Figure; SDMT: Symbol Digit Modalities Test. Statistically significant p-values after FDR correction are marked with *.
