# Supplementary figures and images for: Cognitive profile in multiple sclerosis and post-COVID condition: a comparative study using a unified taxonomy
Source: Sci Rep. 2024 Apr 29;14:9806. doi: 10.1038/s41598-024-60368-0 (PMC11059260; doi:10.1038/s41598-024-60368-0)

A. Time of SARS-CoV-2 infection leading to PCC

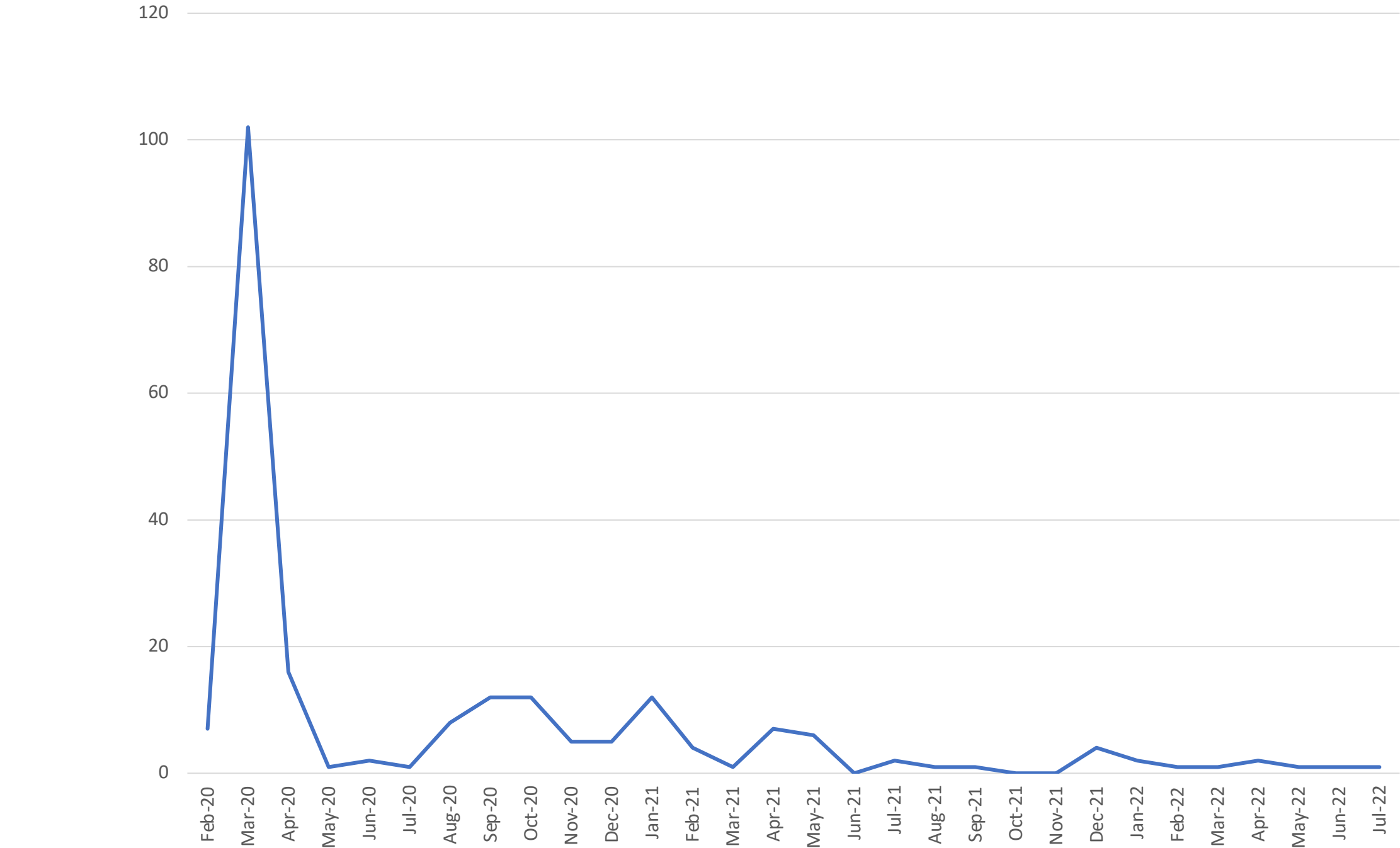

**B. Time of assessment**

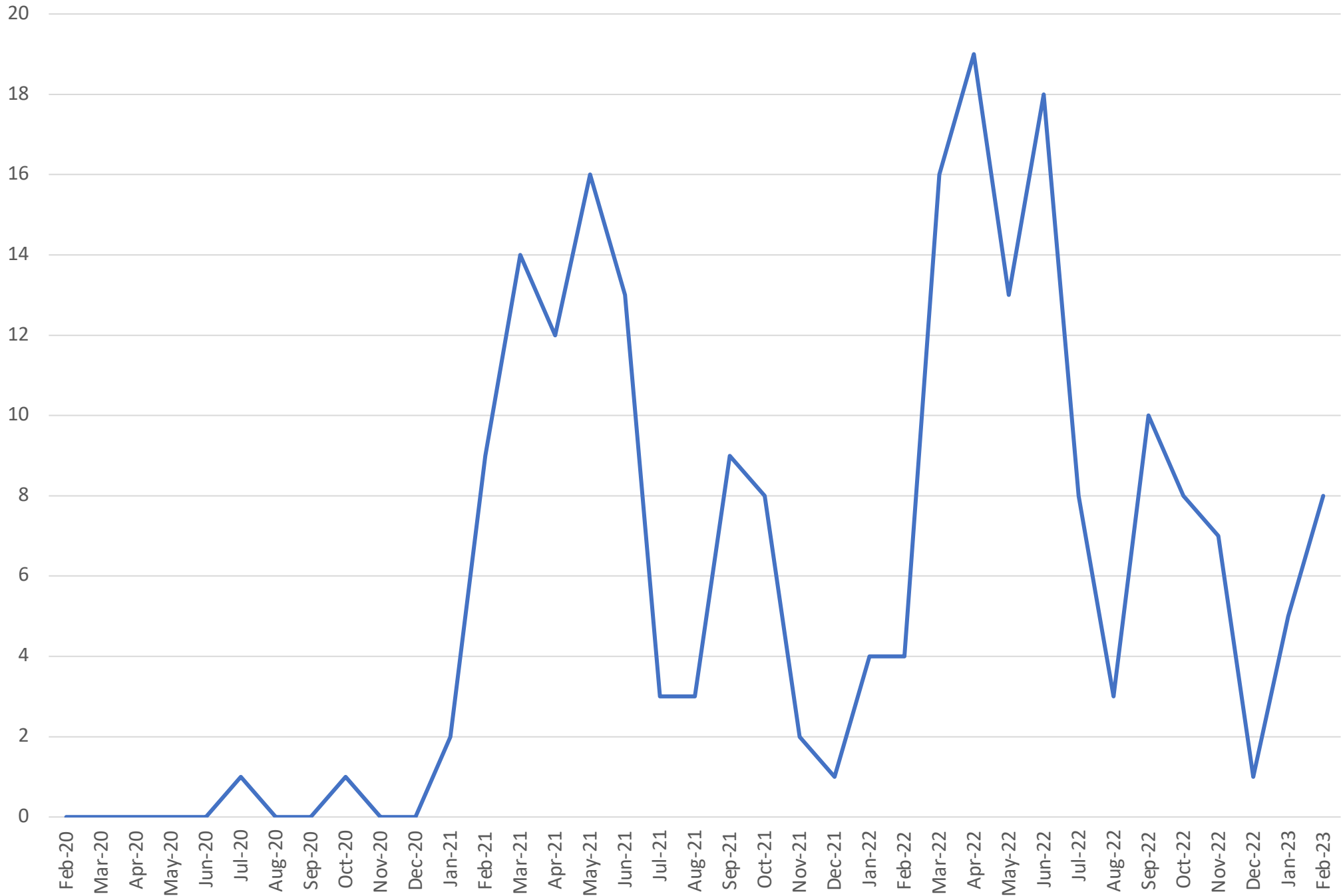

Supplement: Supplementary file 3 — Supplementary Figure 1. [file 41598_2024_60368_MOESM3_ESM.pdf]
