# Supplementary material for: Cognitive profile in multiple sclerosis and post-COVID condition: a comparative study using a unified taxonomy
Source: Sci Rep. 2024 Apr 29;14:9806. doi: 10.1038/s41598-024-60368-0 (PMC11059260; doi:10.1038/s41598-024-60368-0)
Supplement: Supplementary file 4 — Supplementary Figure 2. [file 41598_2024_60368_MOESM4_ESM.pdf]

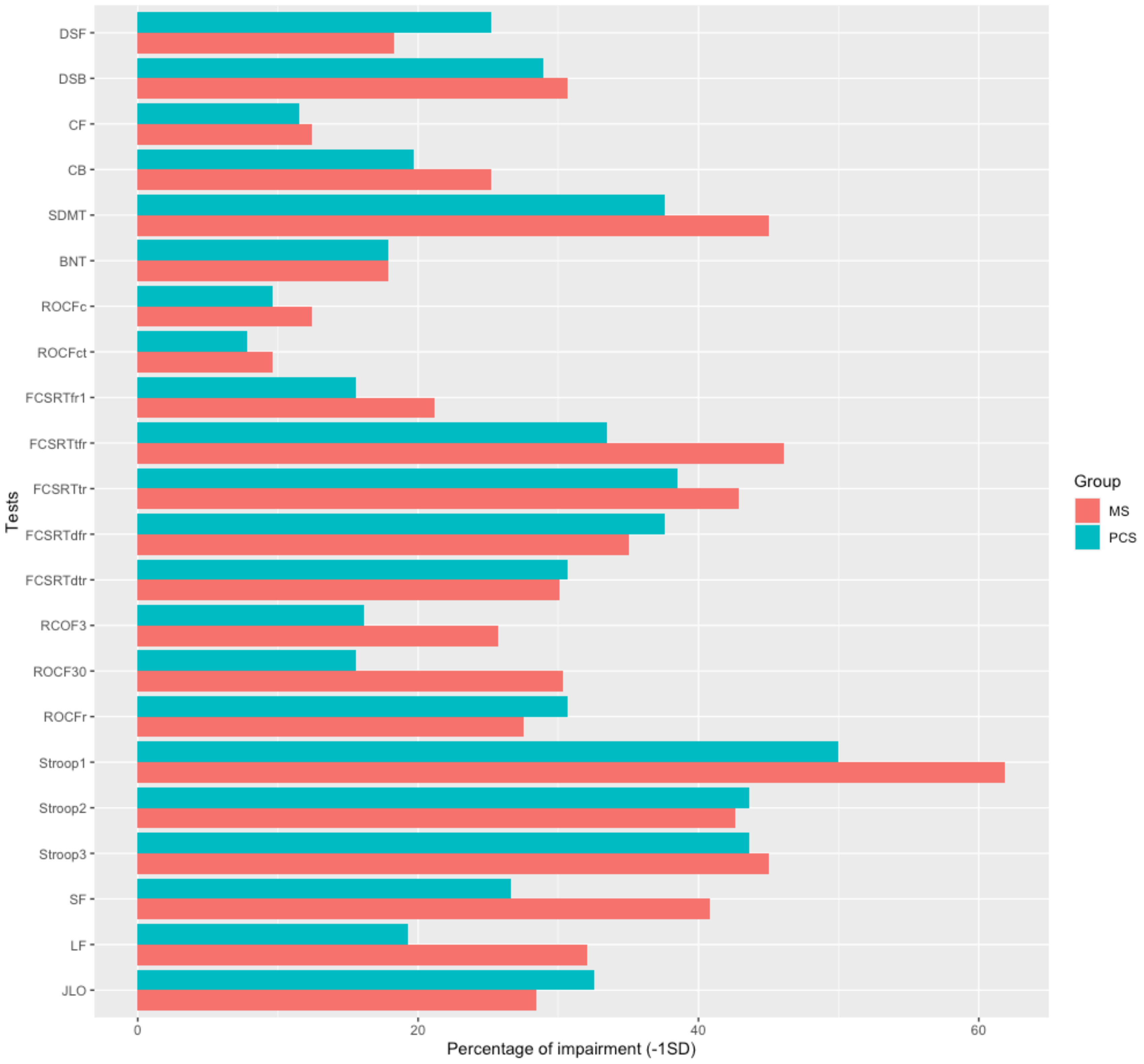

Tests
DSF
DSB
CF
CB
SDMT
BNT
ROCFc
ROCFct
FCSRTfr1
FCSRTtfr
FCSRTtr
FCSRTdfr
FCSRTdtr
RCOF3
ROCF30
ROCFr
Stroop1
Stroop2
Stroop3
SF
LF
JLO
0
20
40
60
Percentage of impairment (-1SD)
Group
MS
PCS
